# Utilisation of monoclonal antibodies in the single radial immunodiffusion assay to determine potency and stability for seasonal and pandemic influenza vaccines

**DOI:** 10.3389/fimmu.2025.1696191

**Published:** 2025-11-17

**Authors:** Jesse Bodle, David Burge, Gopal Gounder, Kirsten Vandenberg, Karen Laurie, Steven Rockman

**Affiliations:** 1 Manufacturing, Science & Technology Dept. (MS&T), CSL Seqirus Ltd, Melbourne, VIC, Australia; 2 Department of Immunology and Microbiology, University of Melbourne, Melbourne, VIC, Australia

**Keywords:** vaccine potency, potency test, monoclonal antibody, mAb, haemagglutinin, influenza vaccine, single radial immunodiffusion (SRID) assay, antigen stability

## Abstract

**Introduction:**

Following manufacture, influenza vaccines are required to undergo potency testing as part of the release process to market. The single radial immunodiffusion (SRID) assay is currently the compendial standard for determining the antigen potency and stability of protein-based influenza vaccines. This assay relies on polyclonal antibodies from animal serum to bind protein antigens in a gel, resulting in a visible precipitin ring. The diameter of this ring is then compared to a calibrated standard to quantify potency. However, the production of polyclonal antiserum introduces delays, impacting the timely release of both seasonal and pandemic vaccines. There is a need for alternative methods that can expedite vaccine release without compromising accuracy or reliability.

**Methods:**

Monoclonal antibodies (mAbs) specific to influenza haemagglutinin (HA) proteins were generated and characterised. Particular mAbs were identified for their ability to react with a broad range of influenza virus strains across different years and seasons, despite antigenic drift and periodic vaccine updates. These mAbs were prepared in advance of the emergence of dominant virus clades, enabling improved pandemic preparedness. In this study, a blend of two mAbs—each targeting distinct regions of the HA protein (the globular head and the stem)—was utilised in an SRID assay format. The assay’s performance was benchmarked against the traditional polyclonal animal sera-based SRID assay for both potency and stability measurements of seasonal and pandemic influenza vaccines.

**Results:**

The combination of two mAbs with alternate specificity enabled the successful precipitation of antigen in the SRID assay. The results obtained from the mAb-based SRID assay demonstrated strong correlation with those from the standard polyclonal sera-based assay. Both potency and stability assessments for various influenza virus samples—including seasonal and pandemic strains—showed comparable outcomes between the two assay formats. The use of specific mAbs allowed for consistent reactivity across multiple influenza seasons, regardless of antigenic drift.

**Discussion:**

This study demonstrates that a mAb-based SRID assay is a viable alternative to the conventional polyclonal sera-based approach for influenza vaccine potency and stability testing. The capacity to generate and stockpile mAbs prior to the dominance of specific virus clades offers the potential for rapid assay deployment, thereby reducing the time required for vaccine release, particularly in pandemic scenarios. Importantly, the adoption of mAb-based assays may not necessitate extensive clinical trials, further expediting the process. Overall, by utilising a carefully selected and well-characterised mix of monoclonal antibodies, this strategy has the potential to substantially improve preparedness and response to emerging influenza threats while upholding rigorous quality standards.

## Introduction

Vaccination is considered the best, most cost-effective defence against influenza virus infection (1). Determining vaccine potency is crucial to ensuring that a vaccine will be effective against its target. For some infectious agents such as polio, the vaccine strain does not need updating due to the stabilised viral genome and significant cross-reactivity of reagents used to determine potency (2). Conversely, for other infectious agents like influenza or SARS-CoV-2 viruses, vaccines undergo regular update to ensure match to circulating strains. Antigenic drift drives amino acid changes primarily in the surface haemagglutinin (HA) protein of the influenza virus. Amino acid changes in influenza HA occur primarily in antigenic sites on the globular head of the protein, which are sites of significant neutralising antibody response (3–7). Alongside, reagents for potency also require update to ensure relevance (8, 9). Upon emergence of a novel influenza virus with pandemic potential, the speed at which vaccine doses are available is essential to mitigating the health impact on the human population (10). This would include the development, manufacture and fill/finish, potency testing and quality testing of vaccines for release.

The compendial assay for determination of influenza vaccine potency is the single radial immunodiffusion (SRID) assay has been in use for over four decades (9, 11). Polyclonal antiserum is immobilised in an agarose gel and antigen (influenza hemagglutinin (HA) within vaccine, or within a reference standard) is introduced into a well punched in the gel and allowed to diffuse. A visible precipitin zone is formed at the point of antigen:antibody equilibrium and the diameter of the zone is measured. Optimisation of both the antigen and antibody concentrations is necessary for adequate precipitin zones. In general, higher antibody concentration leads to smaller diameter zones, while excessively low concentrations lead to poor zone resolution and sensitivity (12). Conversely, the greater the concentration of antigen applied the greater the diameter precipitin zone observed (9). To determine a vaccine’s potency, the diameter of the vaccine zone is compared to a linear calibration curve of a strain-matched HA reference standard of known potency. Reference standard antigen is prepared from pilot or large-scale manufacture. Polyclonal antiserum is generated by immunisation of animals with purified HA protein and adjuvant, which typically requires multiple doses to ensure adequate antibody titres (13). The development and calibration of these antigen and antisera reagents takes between 10 and 12 weeks (1, 9, 13, 14). This includes producing the HA protein immunogen (HA purified from virus or recombinant HA protein) (1–2 weeks), the immunisation schedule in animals, typically sheep, and preparation of antiserum (8–12 weeks), preparation of the reference antigen standard and calibration of the antiserum/reference antigen standard reagents for their use in the compendial assay (1–2 weeks). Upon update of any component of the trivalent (or quadrivalent) influenza vaccines (TIV, QIV), a new antigen/antiserum reagent pair is generated and calibrated.

Following the 2009 influenza A(H1N1) pandemic, Health Canada, the FDA, and the WHO held a workshop in Ottawa to review lessons learned, which included advances to potency assays for rapid vaccine release (14). Subsequent workshops have focused on improvements to the SRID assay and alternate methods to determine influenza vaccine potency and stability (15). Antibody independent and dependent assays have been investigated (15). We have demonstrated that the antiserum reagent can be used to determine potency to antigenically drifted strains in updated seasonal flu vaccines, whereby the use of a heterologous antiserum provides the same calibration value as a homologous antiserum, significantly increases the utility of the reagent (13). We, and others, have developed alternative potency assays that correlate with SRID, utilising haemagglutination-inhibiting (HAI) mAbs in both capture/detection ELISA and SPR technology (16–21) and via a Vaxarry platform (15, 22–24). The benefit of mAbs is the potential for targeted reactivity to antigenically drifted strains over extended periods, covering multiple seasons. The ability to use polyclonal antiserum or mAbs over many seasons reduces the time to availability of a calibrated potency release test, potentially enabling more rapid release and availability of influenza vaccines for administration.

Given the utility of mAbs and longevity of reactivity, there is benefit in understanding the capability of HA-specific mAbs in the compendial SRID potency assay. This study describes use of a mixture of two, or more, mAbs for determining the potency of influenza vaccines. Potency and stability for both seasonal and pandemic influenza vaccines are indicated as proof-of-concept of this strategy.

## Materials and methods

### Monoclonal antibodies

mAbs were produced as previously described (17) using density gradient purified, whole inactivated or live influenza virus propagated in embryonated chicken eggs as immunogens. Antibodies raised against influenza hemagglutinin used in this study are listed in **Supplementary Data**. Antibody reacting to the Influenza A matrix protein was raised against a series of 252 overlapping 15mer peptides (PEPperPRINT™, Heidelberg, Germany) (AMX171.9F5.7E9). The resulting hybridomas were screened and selected based on the method previously described (16). mAbs targeting the stem-region (HA2 sub-unit) of HA (Group 1, CR8020 and Group 2, CR6261) were purchased from Absolute Antibody (Vector Laboratories, CA, USA).

### SRID reagents

Sheep polyclonal anti-HA antisera (As) and strain matched inactivated influenza reference antigen (RA), standardised for HA potency, were obtained from the Therapeutic Goods Administration (TGA, Woden, ACT, Australia), the National Institute for Biological Standards and Control (NIBSC, Potters Bar, Hertfordshire, UK), or the Centre for Biological Evaluation and Research (CBER, Rockville, MD, USA). These reagents were raised against egg or cell-derived strains: A/Victoria/4897/2022-Like (H1N1: TGA AS454 As & 2023/146B RA), A/Thailand/8/2022 (H3N2: TGA AS450 As & 2023/145B RA), A/Kansas/14/2017 (H3N2: TGA AS433 As & 2019/128B RA), A/Thailand/08/2022 (H3N2: TGA AS450 As & 2023/145B RA), A/Tasmania/503/2020 (A/Cambodia/e0826360/2020-like) (H3N2: TGA AS444 & CBER H3-Ag-2107 RA), A/South Australia/55/14 (H3N2: TGA AS408 As & 2014/102B RA), A/Hong Kong/4801/14 (TGA AS412 As & 2016/109B RA), A/North Carolina/04/16 (H3N2: NIBSC 18/108 As, H3-Ag-1801 RA), A/Indiana/08/18 (NIBSC 19/152 As, CBER H3-Ag-1904 RA), A/South Australia/34/19 (TGA AS437 As & 2019/129B RA) and A/Anhui-Lujiang/39/2018 (NIBSC 08/202 As & CBER H9-Ag-2211 RA).

### Influenza vaccines

Monovalent influenza vaccine (MIV) drug substance (DS) was from the Seqirus™ Afluria ^®^ (egg-derived, Melbourne, VIC, Australia) and Flucelvax^®^ (MDCK cell-derived, Holly Springs, NC, USA) vaccine manufacturing platforms as previously described (16). Trivalent influenza vaccines (TIV) were formulated at research scale according to standard SRID potencies to contain no less than 15 micrograms HA per strain per dose (30 μg/mL) representing Influenza subtypes; H1N1(A/Victoria/4897/2022), B-Victoria lineage (B/Austria/1359417/2021) and differing H3N2 components (A/Kansas/14/2017 (clade 3C.3a, TIV1), A/Thailand/08/2022 (clade 2a.3a, TIV2), A/Tasmania/503/2020 (clade 1a, TIV3), and A/Hong Kong/4801/14 (clade 3C.2a, TIV4).

### Forced degradation of HA antigen

Influenza DS and formulated TIV were subject to stress tests of temperature by incubation at 56˚C for 1 hour or 25˚C for 1 month using a ThermoMixer C (Eppendorf South Pacific Pty. Ltd., NSW, Australia) and MIR-154-PE incubator (PHCbi, Tokyo, Japan), respectively. Oxidation involved spiking concentrated DS with hydrogen peroxide solution (Unilab scientific, Kampong Ubi, Singapore) to achieve a final concentration of 2% (v/v), incubating it at room temperature for 1 hour, followed by immediate formulation and testing by SRID.

### HA potency by SRID assay using polyclonal sheep antisera

SRID assays were performed as previously described (25). Briefly, reference antigen and test antigen materials, at equivalent starting concentrations, were diluted 1:1, 2:3 and 1:3 (v/v) in PBS containing 1% (w/v) Zwittergent solution (Calbiochem, Darmstadt, Germany), and added to duplicate wells of agarose gels containing the indicated polyclonal antiserum (SRID reagents). Gels were incubated between 18–24 hours in humidified chambers, dried onto gel bond film (Lonza, Basel, Switzerland), and stained with Coomassie brilliant blue R-250 (Bio-Rad, California, USA). Circular zones of antigen-antibody precipitation were scanned using a Perfection V800 Photo scanner (Epson, Nagano, Japan) and measured using Immulab v12.1.0 software (Microvision instruments, Evry, France). HA concentrations were calculated by parallel line bioassay in comparison to purified inactivated, whole virion reference antigen (26), and test validity was confirmed using a ‘ g ‘ test (g ≤ 0.061) (27).

### HA potency by mAb SRID

mAb SRID was performed as described above with the following modifications. mAbs were diluted to a final concentration of 5 μg/mL antibody in 2% ME agarose (Lonza, Basel, Switzerland) before pouring and setting on gel-bond film. 5ug/ml of mAb was identified in preliminary assays by titrating combinations of globular head mAbs with stem mAb to achieve clear, well defined precipitin zones with diameters typically greater than 5 mm (data not shown). When multiple mAbs were utilised, a total of 5 μg/ml mAb were used in the SRID, whereby mAbs were combined in equal proportions (i.e. 1:1, 1:1:1) at a final concentration of 2.5 μg/ml each when two mAbs were used, at 1.67 μg/ml each when three mAbs were used and at 1.25 μg/ml each when four mAbs were used.

### Statistics

Potency was compared between values determine using SRID with polyclonal sheep antiserum to mAbs by Student’s T-test, using GraphPad Prism (v. 9.3.1) software.

## Results

### A mixture of haemagglutination inhibiting and anti-HA stem mAbs enables detection of HA antigen in the SRID assay

**Figure 1:**
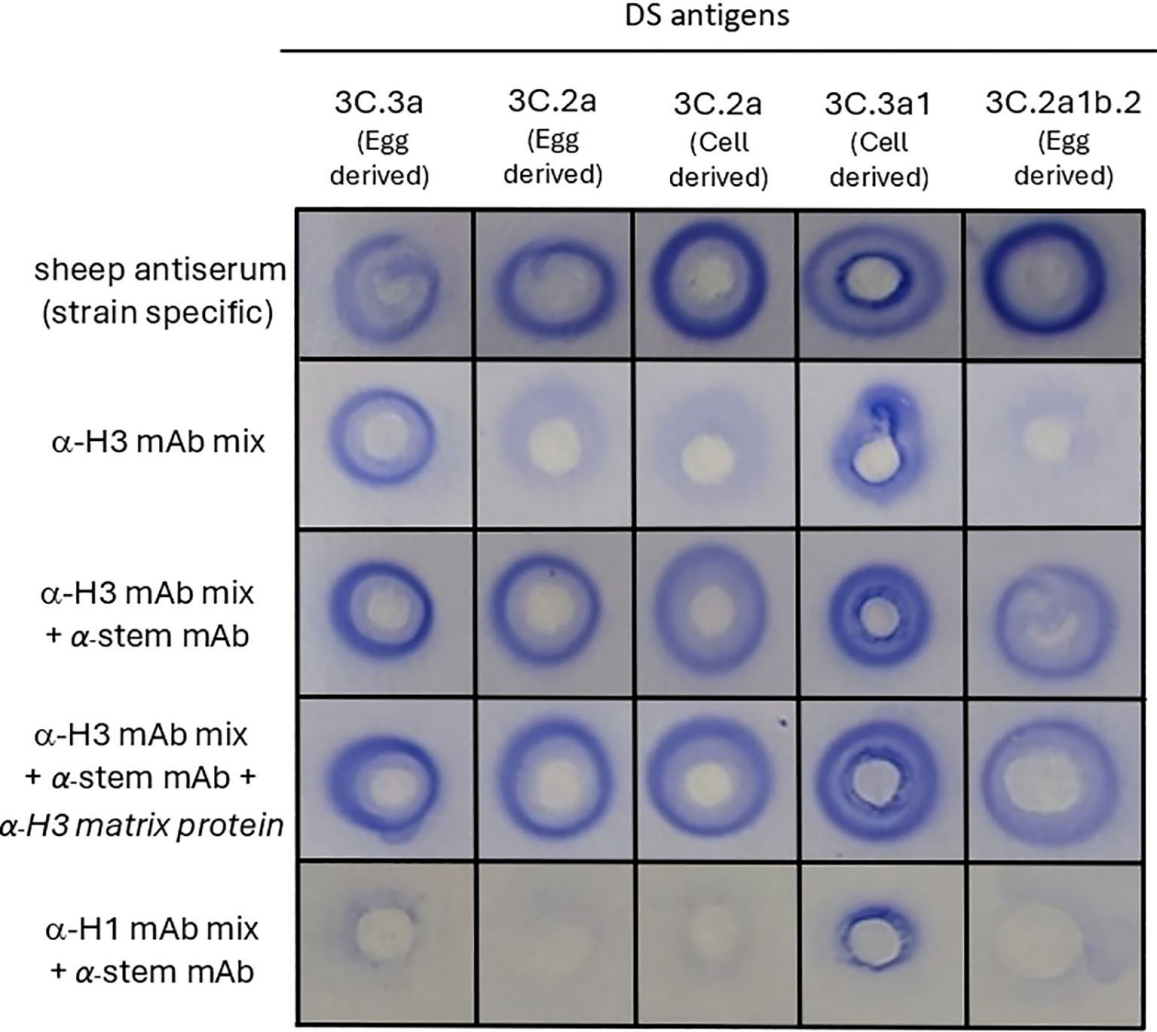
mAbs targeting different regions of HA proteins are required to detect influenza HA antigen in vaccine samples in the SRID assay. Various A(H3N2) antigens (Drug Substance (DS) -egg-derived and cell-derived) belonging to the indicated genetic clades were tested in SRID assays. Agarose was set with sheep antiserum (specific for each genetic clade), or indicated combinations of mAbs, comprised of an anti-H3 mAb mix specific for the globular head (α-H3 mAb mix consisting of mAb - ASIN178.10G10.21F4, HIR146.9E5.1G10, AVIC142.8E5.3E9, TAS160.7E8.1C4 and TAS160.8E7.1B5), an anti-HA2 stem mAb (α-stem mAb – CR8020), anti-H3 matrix mAb (α-H3 matrix protein AMX171.9F5.7E9) and an anti-H1 mAb mix specific for the globular head (α-H1 mAb mix consisting of TORA184.9G2.21B11, SYD198.10F11.24C7, CAL189.3B5.2F4, CAL2.5C6.1E3).

Based on the principles of the SRID assay and literature accounts examining the use of mAbs for antigen quantification (28, 29), we hypothesised that multiple mAbs were needed for cross-linking between antigens and antibodies, to enable precipitation. To test this, a mix of five anti-H3 mAbs specific for the globular head region of HA (anti-H3 mAb mix) and a single mAb specific for the stem region of HA (anti-H3 stem mAb) were tested in SRID assays with various A(H3N2) vaccine antigen candidates (**Figure 1**). The five mAbs in the anti-H3 mAb mix had reactivity to the range of A(H3N2) vaccine antigen candidates; two mAbs with reactivity to all antigens, two mAbs with reactivity to 3C.3a antigens and one mAb with reactivity to 3C.2a antigens (**Supplementary Table**). Whilst the SRID assay using only the anti-H3 mAb mix resulted in a precipitation zone for one out of five A(H3N2) antigens tested (clade 3C.3a), the addition of an anti-stem mAb to the anti-H3 mAb mix demonstrated robust, well-defined precipitation zones for all five A(H3N2) antigens tested (**Figure 1**). Precipitation zones were formed for antigens from various genetic clades regardless of host cell type or manufacturing platform (egg-derived A(H3N2) clade 3C.3a, 3C.2a and 3C.2a1b.2 antigens, cell-derived 3C.2a and 3C.3a1 antigens). The zones were of similar size and intensity to those observed for the strain-specific sheep antisera controls that are distributed by regulatory agencies to determine commercial influenza vaccine potency (**Figure 1**). Combination of the anti-H3 stem mAb with a mix of anti-H1N1 mAbs specific for the globular head of H1 HA (anti-H1 mAb mix), did not result in zonal precipitation, indicating that the anti-H3 stem mAb alone, and mAbs raised against H1 HA globular head, did not facilitate zonal precipitation of the H3 HA antigen. The inclusion of an anti-influenza A matrix protein mAb did not influence the quality, size, or range of reactivity observed (**Figure 1**). These data indicate that a mixture of mAbs, targeting various regions of the HA protein, enables detection of HA in the SRID assay.

### Use of only two mAbs, targeting different epitopes, detect HA antigen in the SRID assay

We next assessed the minimum number and specificities of mAbs required for antigen precipitation in the SRID assay. Three of the five mAbs from the anti-H3 mAb mix (**Figure 1**), were combined with the anti-stem mAb and tested by SRID against a TIV preparation containing an egg-grown 3C.3a antigen (like that utilised in **Figure 1**). Two mAbs had reactivity to all antigens and one mAb had reactivity to 3C.3a antigen (**Supplementary Table**). A combination of two anti-globular head mAbs and the anti-stem mAb were able to generate precipitation zones for the TIV 3C.3a antigen when prepared in a TIV, with similar sized zones as the matched sheep reference antiserum, AS433 (**Table 1**, 36-38 μg HA/ml when detected by mAbs and 39 μg HA/ml when detected by AS433 for TIV 1). When the combination of mAbs was further reduced to a single anti-globular head mAb and anti-stem mAb, precipitin zones were also observed at similar sizes to the reference antiserum, AS433 (**Table 1**, 36-42 μg HA/mL detected by mAbs and 39 μg HA/ml when detected by AS433 for TIV 1). The mAbs were assessed against a panel of TIVs (TIV 2- 4), differing solely by the H3N2 strain incorporated. The four A(H3N2) strains selected represent clades 3C.3a, 2a.3a, 1a, and 3C.2a, isolated between 2014 and 2022, spanning a nine-year period.

**Table 1:** Mixes of two or three mAbs may be used in the SRID assay to detect influenza HA antigen in vaccine samples.

mAb or sheep antiserum used in SRID assay		Number of mAbs in mix	Potency of H3 HA by SRID (μg HA/mL)			
			TIV1 H3N2 clade 3C.3a	TIV2 H3N2 clade 2a.3a	TIV3 H3N2 clade 1a	TIV4 H3N2 clade 3C.2a
mAb mix	α-H3 mAb 1 + α-H3 mAb 2 + α-stem mAb	3	36	43	42	39
	α-H3 mAb 1 + α-H3 mAb 3 + α-stem mAb	3	36	39	40	36
	α-H3 mAb 2 + α-H3 mAb 3 + α-stem mAb	3	38	39	39	34
	α-H3 mAb 1+ α-stem mAb	2	36	44	39	40
	α-H3 mAb 2 + α-stem mAb	2	42	35	39	39
	α-H3 mAb 3 + α-stem mAb	2	38	BLD	BLD	BLD
Sheep Antiserum Reagent (genetic clade)	AS433 (3C.3a)	n/a	**39**	BLD	49	43
	AS450 (2a.3a)	n/a	38	**39**	54	BLD
	AS444 (1a)	n/a	44	24	**34**	40
	AS412 (3C.2a)	n/a	39	BLD	36	**35**

BLD; Below Limit of Detection. n/a: not applicable.

TIVs were formulated using reference antigens to contain A(H1N1), various A(H3N2) and influenza B/Victoria strains targeted at 38 μg HA/mL/strain. A(H3N2) component varied for each TIV: genetic clades 3C.3a (TIV1), 2a.3a (TIV2), 1a (TIV3), and 3C.2a (TIV4). Agarose was set with control sheep antiserum (specific for each A(H3N2) strain, results highlighted in bold), or combinations containing an anti-HA2 mAb (α-stem mAb – CR8020) and mAbs targeting the globular head (α-H3 mAb 1 ASIN178.10G10.21F4, α-H3 mAb 2 - HIR146.9E5.1G10, α-H3 mAb 3 - TAS160.8E7.1B5). Potencies for A(H3N2) strains were determined using strain matched CBER or NIBSC reference antigen.

Mixtures of mAbs containing two anti-globular head mAbs and the anti-stem mAb demonstrated strong cross-clade reactivity, resulting in accurate potency calculations for all four TIV formulations tested. The mean accuracy (% expected vs. observed) was 99% (N=12), ranging between 88% and 112%. Mixtures containing one anti-globular head mAb and one anti-stem mAb were also cross-reactive, with anti-H3 mAbs 1 and 2 forming precipitin zones for all virus clades when mixed with an anti-stem mAb. Anti-H3 mAb 3 was specific for 3C.3a HA only, suggesting clade specificity despite inclusion of the (within sub-type) anti-stem mAb in the mixture. The mean accuracy (% expected vs. observed) reported for a mixture of two mAbs (one anti-globular head mAb and one anti-stem mAb) was 98% (N=10), ranging between 86% and 109%. Interestingly, while sheep antisera could be used in a heterologous format to determine a potency, in this experiment the accuracy was inconsistent ranging between 70% and 158%. Taken together, these data indicate the simplicity and effectiveness of this approach of a mix of mAbs for potency measurement over many seasons of influenza circulation.

### Demonstration of potency and stability of seasonal influenza vaccine drug substance and drug product using a mix of mAbs in the SRID assay

The SRID assay is primarily used to evaluate the potency of monovalent influenza antigen drug substance (DS) and the potency and stability of final formulated TIV or quadrivalent (QIV) influenza vaccines. When monovalent DS and TIV containing A(H1N1), A(H3N2) and B/Victoria DP formulations were assayed for potency alongside calibrated reference standards, similar potencies (no statistical difference, p>0.05) were observed for H1 HA and H3 HA in the DS and TIV samples, when assays were performed using a mix of mAbs as compared to polyclonal sheep antisera (**Table 2**). For all vaccines, a potency assay is required to determine the active antigen that is to be delivered to the recipient, as well as evaluate potency with respect to stability. Stability analysis can be performed by incubating the vaccine material at the required storage temperature over time or can also be assessed by incubation at increased (‘accelerated’) temperatures (30). To assess the utility of mixtures of mAbs in the SRID assay to indicate stability, DS and TIV were heated at 56°C and 25°C respectively, prior to testing in the SRID assay. mAbs used were predominantly directed to non-confirmational epitopes (**Supplementary Data**). After incubation at accelerated temperature, both DS & TIV samples reported similar potencies for H1 and H3 antigens with no statistical difference compared to antisera controls (**Table 2**, p>0.05). Under both accelerated temperature conditions, SRID measured using mAbs showed reduced potencies, consistent with the reductions observed when using SRID measured with polyclonal antisera (**Table 2**, DS at 56°C: 68% reduction in potency (mAb) vs. 64% (antisera) for H1 and 70% reduction in potency (mAb) vs. 79% (antisera) for H3. TIV at 25°C: 23% reduction in potency (mAb) vs. 32% (antisera) for H1 and 14% reduction in potency (mAb) vs. 33% (antisera) for H3). Taken together, this data indicates that use of mAbs in the SRID assay is potency and stability indicating.

**Table 2:** Assessment of mixtures of mAbs to determine potency and stability of DS and TIV formulations.

Antigen	Subtype detected	Sheep antisera	α-H1 or α-H3 mAb mix	# of mAbs in mix
		SRID (μg HA/mL)		
Monovalent DS; 2-8°C	A(H1N1)	1359	1077	5
	A(H3N2)	1667	1685	5
Monovalent DS: 56˚C/1 Hr.	A(H1N1)	492	349	5
	A(H3N2)	342	500	5
TIV5; 2-8°C	A(H1N1)	31	22	5
	A(H3N2)	40	35	5
TIV5: 25˚C/1 Mth.	A(H1N1)	21	17	5
	A(H3N2)	27	30	5
% Reduction in potency*				
Monovalent DS: 56˚C/1 Hr.	A(H1N1)	64	68	
	A(H3N2)	79	70	
TIV5: 25˚C/1 Mth.	A(H1N1)	32	23	
	A(H3N2)	33	14	

*% Reduction in potency:

Monovalent DS=(1-(56 ˚C/2-8 ˚C control) * 100).

TIV=(1-(25 ˚C/2-8 ˚C control) * 100).

Monovalent drug substance (DS) and TIV (TIV2) held at 2-8°C post manufacture/formulation, 56˚C for 1 hour or 25˚C for 1 month, were assessed for potency in SRID comparing mixtures of mAbs to sheep polyclonal antisera reagents. α-H1 mAb mix contained globular head-specific mAbs: TORA184.9G2.21B11, SYD198.10F11.24C7, CAL189.3B5.2F4, CAL2.5C6.1E3 and HA2-specific mAb: CR6121, α-H3 mAb mix contained globular head-specific mAbs: ASIN178.10G10.21F4, HIR146.9E5.1G10, THA202.9G5.31C3, THA202.3F7.13G6 and HA-2-specific mAb: CR8020, Sheep polyclonal antisera (TGA: A(H1N1) - AS454, A(H3N2) - AS450.

### Demonstration of potency and stability of pre-pandemic influenza vaccine DS using a mix of two mAbs in the SRID assay

**Figure 2:**
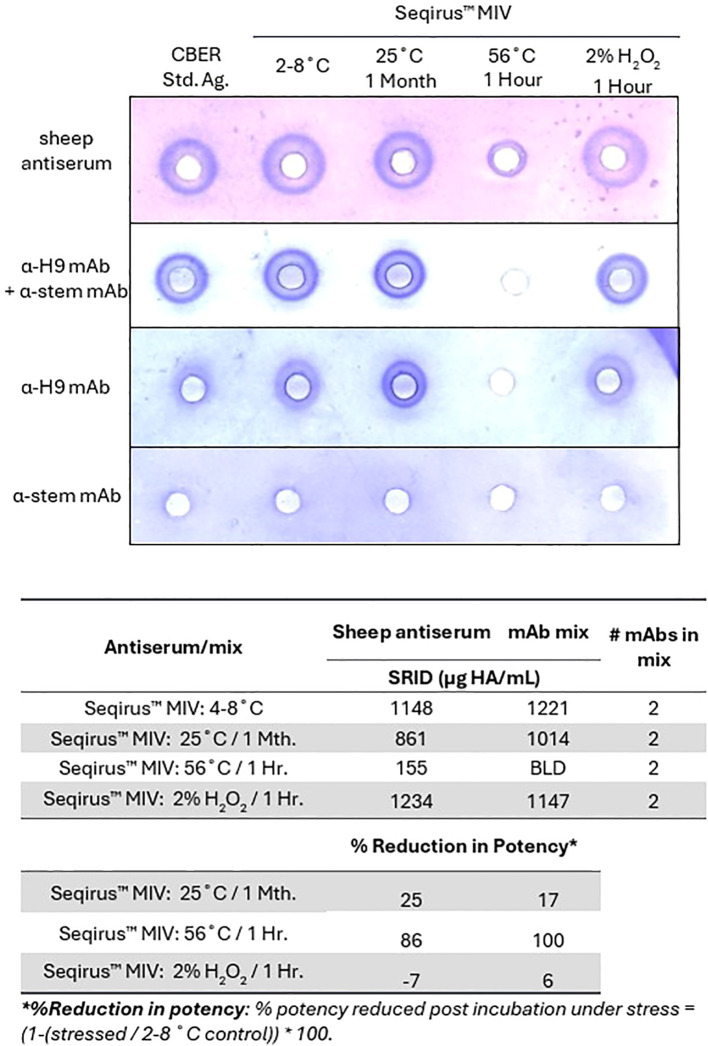
Two mAbs may be used in SRID to measure potency and stability of a zoonotic influenza HA antigen in DS. A(H9N2) antigen (MIV DS), stored at typical conditions (2-8°C), or under accelerated temperature (25°C for 1 month or 56°C for 1 hour) or oxidative conditions (2% H2O2 for 1 hour), were tested in a SRID assay, alongside a calibrated reference antigen (CBER: H9-Ag-2211) stored at typical conditions (2-8°C). Agarose was set with virus-specific sheep antiserum (NIBSC 08/202), an anti-H9 mAb specific for the globular head of HA (α-H9 mAb, AL207.2G8.25B6), an anti-HA2 mAb (α-stem mAb, CR6261) or a mixture of α-H9 mAb and α-stem mAb. Quantitative assessment of SRID precipitation zones measured using sheep antisera or the mix of α-H9 mAb and α-stem mAb.

To understand the impact of this strategy on preparedness for pandemic influenza vaccines, the use of mAbs within SRID were tested against a zoonotic DS, from a pre-pandemic candidate vaccine virus (CVV). A single anti-globular head mAb and a single anti-stem mAb were mixed and used in SRID to assess an A(H9N2) monovalent influenza vaccine (MIV) preparation for potency and stability. As for seasonal DS and DP, the mAb combination induced strong zones for the MIV stored at standard conditions of 2-8°C, with high correlation of potency to the control sheep polyclonal antiserum (**Figure 2**). Use of the anti-H9 globular head mAb alone resulted in a visible zone, although of reduced quality (**Figure 2A**), making quantitation unachievable.

Stability was assessed using samples incubated under accelerated temperature and oxidative stress, which were expected to denature the HA trimer (**Figure 2B**). As was observed with seasonal strains, the size of zones generated by the mix of mAbs correlated well with those generated by sheep antiserum for most samples. Under accelerated temperature incubation at 25°C for 1 month, SRID measured using mAbs reported reduced potencies, consistent with the reductions observed using SRID measured with polyclonal antisera (17% reduction in potency mAb-SRID vs. 25% sheep antisera). Interestingly, significant degradation was detected in the sample incubated at 56°C for 1 hour by sheep antiserum (86% reduction of potency reported), whilst no zone was detected using the blend of mAbs (100% reduction in potency reported). No notable reduction in potency was observed with either antibody preparation after treatment under oxidative conditions, however a high correlation in reported potencies was maintained between mAb-SRID and standard SRID using sheep antisera (108%).

## Discussion

This study demonstrates that two mAbs are sufficient to accurately measure the potency of influenza HA in a SRID assay. The mAbs must be specific for alternate epitopes, such as the globular head and the HA2 stalk region of the HA protein. This mAb-based SRID assay aligned with the compendial SRID assay using polyclonal antisera for measurement of stability, as well as potency, of monovalent DS or TIV. mAbs that offer broad cross-reactivity to antigenically drifted A(H3N2) viruses are applicable to this assay, demonstrating the longevity of this approach. The availability and rapid generation of mAbs for use in the SRID assay provides opportunity for quicker availability of influenza vaccines to prevent disease.

Timelines for current seasonal and pandemic influenza vaccine release are dictated by the generation and calibration of reference reagents for the SRID assay (1, 9, 14). Motivated by learnings from the 2009 influenza pandemic, international workshops over the past 15 years have explored alternatives to the SRID assay using polyclonal sheep antiserum (14, 15). Many groups, including our group, have assessed antibody-based (ELISA, SPR), and antibody-independent methods (RP-HPLC, IDMS) to measure vaccine potency and stability (14, 15, 19, 21–24, 31–33). Most recently the method used to calibrate the HA mass for the SRID reference antigen was updated to IDMS from gel electrophoresis (34). There are multiple challenges for implementation of alternate potency assays for influenza vaccines. Specific attention should be given to measurement of a biologically relevant potency (functionally active HA), which is stability indicating (35). While some alternative assay methods are proposed to indicate stability, the conditions tested are often more aggressive than typical storage environments which may not accurately represent gradual stability changes throughout a product’s shelf life (26). Further, the unique stability profiles observed for different vaccine matrices assayed using the compendial SRID may not be consistent when using an alternate assay (36, 37). Thus, use of the current SRID assay format with mAbs may be considered as a complementary approach, potentially without the need for clinical trials for current vaccines.

To the best of our knowledge, we have demonstrated for the first time, measurement of potency of influenza vaccines, with only two mAbs using the SRID assay. Marcovina et al. described the use of four mAbs in a double immunodiffusion assay for quantifying human plasma apolipoprotein. Results correlated with those from assays using polyclonal antisera (28, 29). The complementary specificity and reactivity of mAbs is important. A clear benefit of mAbs is the ability to direct reactivity of binding. mAbs may target a conserved region of the HA protein, such as the HA2 stalk. mAbs also may be specific for semi-conserved regions of the HA globular head maintaining broad specificity within an influenza A subtype, or influenza B lineage. These mAbs offer utilisation over many influenza seasons despite vaccine recommendation updates, such as those mAbs used in this study which have reactivity across four A(H3N2) clades isolated between 2014 and 2022 (16, 17). Notably, we have also demonstrated the utility of sheep polyclonal sera in potency assays across multiple years (13). These approaches significantly reduce the use of animals for scientific purposes. mAbs may also be virus/clade specific. There is utility for clade-specific mAbs in SRID assessment of potency of QIV formulations. From 2012 to 2023, QIV formulations included both B/Victoria and B/Yamagata influenza lineages, leading to cross-reactivity with antisera reagents (38). As such, manufacturers were required to assess cross-lineage reactivity of each new antiserum and ensure any cross-reactivity was accounted for in the validation of the potency assays, which increased time for implementation of assays. Following the disappearance of the B/Yamagata lineage in 2020, this lineage was removed from seasonal influenza vaccine formulations, enabling potential improvement to seasonal vaccine by inclusion of a second A(H1N1) or A(H3N2) strain (39). In the case of an additional A(H3N2) clade added to make a QIV, mAbs provide opportunity for specific reagents and assays. One such mAb used in this study (TAS.160.8E7) recognises A(H3N2) 3C.3a viruses, but not viruses from the 3C.2a, 1a or 2a.3a clades, even when mixed with a highly cross-reactive anti-HA2 stem mAb. Further, the choice of mAbs may be targeted to enable potency and stability assessment over many seasons and/or vaccine formulation or valency. These results underscore the need for high-avidity, non-overlapping mAb selection to ensure effective antigen precipitation and minimise competitive or steric interference.

Upon emergence of a novel influenza virus, with pandemic potential, panels of existing mAbs can be screened and new mAbs can be generated. A hybridoma clone can be generated from mice within 7 weeks upon availability of antigen. Significant quantities of purified mAb can be generated after a further 3–4 weeks. During the COVID-19 pandemic, 17 unique hybridoma clones specific for the receptor binding domain of SARS-CoV-2 virus spike protein were available from our laboratory in pure concentrated form within 11 weeks from immunisation. For influenza, with many subtypes identified and isolated, there is opportunity to generate panels of mAbs targeting the globular head of various HA proteins. Panels (of mAbs) may be aliquoted in 96 or 384-well plates for storage, to enable rapid screening of mAbs upon emergence of a novel strain. Like non-seasonal (zoonotic) candidate virus vaccines that are produced by the WHO for pandemic preparedness (40), clonal hybridoma cell lines may be stored at low temperatures. Upon thaw, these clonal cells can be seeded in bioreactors for expansion, mAb purification and concentration to generate larger quantities for use in approximately 3–4 weeks. Unlike generation of polyclonal antisera in sheep where the diversity of each animal antiserum may affect reactivity (13) mAbs offer a stable alternative. In the case of seasonal influenza, new viruses are routinely screened against hybridoma panels to ensure reactivity and new mAbs are generated as needed. Wadey et al., cited the application of cross-reactive polyclonal antisera was maintained between 2 and 14 seasons (13). Based on evidence of our mAbs cross-reacting with strains isolated over a period of 13 years (mAb clone SIN178.10G10) this suggests the utility of this mAb in combination with the anti-stem mAb could be viable for as many as 26 seasons (16).

This study has several limitations. A small number of viruses and mAbs were tested to show feasibility and comparability. Whilst egg and cell grown viruses were assessed, other vaccine matrices have not been included. Further studies may characterise the specificity of the mAbs used to understand the minimal proximity of the two epitopes targeted, to prevent steric hindrance and enable visible precipitation. Furthermore, it would be an advantage to strengthen our understanding of which major antigenic regions of HA1—and how many—should be represented by targeted mAbs within the mAb mix. Targeting the 4 (Influenza B) and 5 (Influenza A) non-overlapping antigenic sites of HA1 globular head (41, 42), may enable broad reactivity whilst retaining accurate representation of stability. This is particularly important with respect to demonstrating stability under mild conditions, as it may enhance the correlation between compendial SRID and mAb SRID assays in these settings. Surprisingly, the A(H9N2) MIV was not denatured when placed under oxidative stress as measured by both mAb and polyclonal antibodies in the SRID assays, Whilst it is possible that insufficient oxidative stress was applied, it has also been reported that reactive oxygen species can promote HA multimerisation, potentially forming higher-order structures. These encapsulated HA trimers would be shielded from oxidation (43, 44). Temperature treatment was sufficient to denature the H9 HA protein, as detected, most likely by the anti-stem antibody which recognises a confirmational epitope. These findings underscore the necessity of assessing multiple non-overlapping globular head mAbs and methods to achieve robust and reliable evaluation of HA stability ensuring an accurate, correlative measurement of potency for HA under stressed conditions. Notably, mAbs generated in our laboratory have reactivity by ELISA against multiple sub-types of HA including: H1, H3, H5, H7 and H9.

The SRID is an accepted assay for measurement of potency and stability of influenza vaccines (9, 13, 25). It is relatively simple to perform and accessible, with no need for expensive equipment. Utilisation of a mixture of mAbs, offers a strategy with likely time, cost and logistical benefits for influenza vaccine release and availability, increasing the use of reagents across seasons and decreasing this use of animals. Given the current use of SRID across all licensed platforms, incorporating mAbs into SRID could potentially reduce or eliminate the need for expensive clinical trials during implementation.

## Data Availability

The raw data supporting the conclusions of this article will be made available by the authors, without undue reservation.
