# Supplementary material for: Utilisation of monoclonal antibodies in the single radial immunodiffusion assay to determine potency and stability for seasonal and pandemic influenza vaccines
Source: Front Immunol. 2025 Nov 17;16:1696191. doi: 10.3389/fimmu.2025.1696191 (PMC12665921; doi:10.3389/fimmu.2025.1696191)
Supplement: Supplementary file 1 [file DataSheet1.pdf]

Supplementary information

Methods for characterisation of mAbs

mAbs were isotyped using the Mouse Isotyping kit (Roche, Basel, Switzerland). mAb subtype and clade reactivity were characterised by direct-coat ELISA as previously described (1). The ability of a mAb to inhibit agglutination of avian and mammalian red blood cells were assayed as previously described (1). mAbs were assessed by western blot (non-reduced SDS-PAGE) to the immunising antigen using Invitrogen iBlot™ system as described in the user manual (Thermo Fisher Scientific, Massachusetts, USA). mAbs raised against H3 HA were binned using a capture sandwich assay approach assessing each mAb in combination with titrating titres of alternative 'blocker' mAbs, against recombinantly generated HA antigen. Binning was analysed by SPR using a Biacore™ 8K+ instrument and CM5 chip (Cytiva, MA, USA). mAb clone HIR146.9E5.1G10 was further mapped via escape mutagenesis as previously described (2).

Supplementary Table 1. Characterisation of mAbs targeting H3 HA. Seven mAbs raised to the globular head domain of influenza H3 HA were characterised by isotype; subtype and clade specificity by direct-coat ELISA; reactivity to linear epitopes by western blot and haemagglutination inhibition (HAI) by binding to RBC. Binding to common epitopes between mAbs was assessed by binning experiments. Identification of the binding epitope was assessed by escape mutagenesis (HIR146.9E5.1G10 mAb only).

mAb (HA immunogen, isotype)	mAb reactivity by ELISA[a]							Western Blot	HAI reactivity	mAb Binning	Figure / Table	Reference
	3C.2A EGG	3C.2A CELL (TIV 4)	3C.3A1 CELL	3C.2A1B.2 EGG	3C.3A EGG (TIV 1)	2a.3a.1 (TIV 2)	1a (TIV 3)					
AVIC142.8E5.3E9 (A/Victoria/563/2010, IgG1κ)	NO	YES	NO	NO	NO	NO	NO	Negative	YES	Not Tested	Figure 1	N/A
TAS160.7E8.1C4 (A/Tasmania/11/2014, IgG1κ)	YES	NO	YES	NO	YES	NO	YES	Negative	YES	COMPLETELY blocked by TAS160.8E7. NOT blocked by HIR146.9e5.1G10	Figure 1	(1)
TAS160.8E7.1B5 (A/Tasmania/11/2014, IgG1κ)	NO	NO	YES	NO	YES	NO	NO	Negative	YES	COMPLETELY blocked by TAS160.8E7. NOT blocked by HIR146.9E5.1G10	Figure 1 Table 1	N/A
ASIN178.10G10.21F4 (A/Singapore/GP2050/15, IgG1κ)	YES	YES	YES	YES	YES	YES	YES	Negative	YES	PARTIALLY blocked by THA202.9G5, THA202.3F7, TAS160.7E8,TAS160.8E7. NOT blocked by HIR146.9E5.1G10	Figure 1 Table.1 Table 2	(1)
HIR146.9E5.1G10 (A/Hiroshima/52/05, IgG2aλ)	YES	YES	YES	YES	YES	YES	YES	Negative	NO	NOT blocked by ASIN178, THA202.9G5, THA202.3F7,TAS160.7E8,TAS160.8E7. Escape Mutagenesis: K276N Epitope C.	Figure 1 Table 1 Table 2	N/A
THA202.9G5.31C3 (A/Thailand/08/2022, IgG1κ)	YES	YES	YES	YES	YES	YES	NO	Positive	YES	COMPLETELY blocked by THA202.3F7,TAS160.7E8,TAS160.8E7. NOT blocked by HIR146.9E5.1G10	Table 2	N/A
THA202.3F7.13G6 (A/Thailand/08/2022, IgG1κ)	YES	YES	NO	NO	NO	YES	NO	Negative	YES	COMPLETELY blocked by THA202.9G5. NOT blocked by HIR146.9E5.1G10	Table 2	N/A

[a] HA antigens for screening: 3C.2a egg – A/Hong Kong/4801/2014; 3C.2a cell - A/North Carolina/04/2016; 3C.3a1 cell – A/Indiana/08/2018; 3C.2a1b.2 egg - A/South Australia/34/19; 3C.3a egg (TIV 1) – A/South Australia/55/2014; 2a.3a.1 (TIV 2) – A/Thailand/08/2022; 1a (TIV 3) – A/Tasmania/503/2020

Supplementary Table 2. Characterisation of mAbs targeting H1 HA. Four mAbs raised to the globular head domain of influenza H1 HA were characterised by isotype; subtype and clade specificity by direct-coat ELISA; reactivity to linear epitopes by western blot and haemagglutination inhibition (HAI) by binding to RBC.

mAb (HA immunogen, isotype)	mAb reactivity by ELISA[a] 5a.2a.1 EGG	Western Blot	HAI reactivity	Figure / Table	References
TORA184.9G2.21B11 (A/Victoria/2454/2019, IgG3κ)	YES	Negative	YES	Figure 1 Table 2	(1)
SYD198.10F11.24C7 (A/Sydney/5/2021, IgG1κ)	YES	Positive	YES	Figure 1 Table 2	N/A
CAL189.3B5.2F4 (A/California/07/2009, IgG1κ)	YES	Positive	NO	Figure 1 Table 2	N/A
CAL2.5C6.1E3 (A/California/07/2009, IgG1κ)	YES	Positive	YES	Figure 1 Table 2	(1,3)

[a] HA antigen for screening– 5a.2a.1 egg – A/Victoria/4897/2022 – Included as representative H1N1 in TIV formulations 1-4.

Supplementary Table 3. Characterisation of mAb targeting H9 HA. One mAb raised to the globular head domain of influenza H9 HA was characterised by isotype; subtype and clade specificity by direct-coat ELISA and reactivity to linear epitopes by western blot.

mAb (HA immunogen, isotype)	mAb reactivity by ELISA[a] Y280/G9 EGG	Western Blot	Figure / Table	References In Literature
AL207.2G8.25B6 (A/Anhui-Lujiang/39/2018, IgG2aκ)	YES	Positive	Figure 2	N/A

[a] HA antigen for screening– Y280/G9 egg – A/Anhui-Lujiang/39/2018

Supplementary Table 4. Characterisation of mAbs targeting stem region of HA group 1 & 2 influenza viruses.

Summary characterisation data from published sources. The ability of the mAbs to inhibit haemagglutination (HAI) was assessed by binding to RBC.

mAb (HA immunogen, isotype)	mAb reactivity by ELISA	Western Blot	HAI reactivity	Epitope Mapping	Figure / Table
CR6261 (Isolated from human memory B cell using phage display (4), IgG1λ)	Broadly neutralising antibody reacts to all group 1 influenza viruses (5). Specific to this study sub-types: H1N1 & H9N2.	Positive(6)	YES	Binds a conserved conformational epitope of the pre-fusion state HA stem region at the membrane-proximal end of HA trimer. The epitope is primarily composed of the HA2 A-helix but also contacts residues from the HA1 stem (7)	Table 2<br>Figure 2
CR8020 (Isolated from human memory B cells, IgG1κ)	Broadly neutralising antibody reacts to all group 2 influenza viruses (4,8). Specific to this study sub-type: H3N2.	Positive(6)	YES	Binds a conserved conformational epitope of the pre-fusion state HA stem region that is partially overlapping with the fusion peptide and close to the cleavage site of HA (9)	Figure 1<br>Table 1<br>Table 2

1. Bodle J, Vandenberg K, Laurie K, Barr IG, Zhang Y, Rockman S. An ELISA-based assay for determining haemagglutinin potency in egg, cell, or recombinant protein derived influenza vaccines. *Front Immunol* (2023) 14:1147028. doi: 10.3389/fimmu.2023.1147028

2. Webster RG, Laver WG. Determination of the number of nonoverlapping antigenic areas on Hong Kong (H3N2) influenza virus hemagglutinin with monoclonal antibodies and the selection of variants with potential epidemiological significance. *Virology* (1980) 104:139–148. doi: 10.1016/0042-6822(80)90372-4

3. Bodle J, Verity EE, Ong C, Vandenberg K, Shaw R, Barr IG, Rockman S. Development of an enzyme-linked immunoassay for the quantitation of influenza haemagglutinin: an alternative method to single radial immunodiffusion. *Influenza Other Resp* (2013) 7:191–200. doi: 10.1111/j.1750-2659.2012.00375.x

4. Ekiert DC, Wilson IA. Broadly neutralizing antibodies against influenza virus and prospects for universal therapies. *Curr Opin Virol* (2012) 2:134–141. doi: 10.1016/j.coviro.2012.02.005

5. Throsby M, Brink E van den, Jongeneelen M, Poon LLM, Alard P, Cornelissen L, Bakker A, Cox F, Deventer E van, Guan Y, et al. Heterosubtypic Neutralizing Monoclonal Antibodies Cross-Protective against H5N1 and H1N1 Recovered from Human IgM+ Memory B Cells. *PLoS ONE* (2008) 3:e3942. doi: 10.1371/journal.pone.0003942

6. Brandenburg B, Koudstaal W, Goudsmit J, Klaren V, Tang C, Bujny MV, Korse HJWM, Kwaks T, Otterstrom JJ, Juraszek J, et al. Mechanisms of Hemagglutinin Targeted Influenza Virus Neutralization. *PLoS ONE* (2013) 8:e80034. doi: 10.1371/journal.pone.0080034

7. Ekiert DC, Bhabha G, Elsliger M-A, Friesen RHE, Jongeneelen M, Throsby M, Goudsmit J, Wilson IA. Antibody Recognition of a Highly Conserved Influenza Virus Epitope. *Science* (2009) 324:246–251. doi: 10.1126/science.1171491

8. Fleishman SJ, Whitehead TA, Ekiert DC, Dreyfus C, Corn JE, Strauch E-M, Wilson IA, Baker D. Computational Design of Proteins Targeting the Conserved Stem Region of Influenza Hemagglutinin. *Science* (2011) 332:816–821. doi: 10.1126/science.1202617

9. Ekiert DC, Friesen RHE, Bhabha G, Kwaks T, Jongeneelen M, Yu W, Ophorst C, Cox F, Korse HJWM, Brandenburg B, et al. A Highly Conserved Neutralizing Epitope on Group 2 Influenza A Viruses. *Science* (2011) 333:843–850. doi: 10.1126/science.1204839
